# Supplementary material for: Modeling the hallucinatory effects of classical psychedelics in terms of replay-dependent plasticity mechanisms
Source: eLife. 2026 Apr 21;14:RP105968. doi: 10.7554/eLife.105968 (PMC13099140; doi:10.7554/eLife.105968)
Supplement: Supplementary file 1. [file elife-105968-supp1.pdf]

Parameter	Function	Value
$\{N^{(l)}\}$	Layer Widths	$[32, 16, 6]$
σ_b	Inference standard dev.	0.01
σ_p	Generative standard dev.	0.01
N_d	Number of dendritic branches	16
L	Epoch number	50
T_w	Length of Wake phase	200
T_s	Length of Sleep phase	200
K	Batch number	512
η_p	Generative parameter learning rate	0.001
η_b	Inference parameter learning rate	0.001
$[\beta_1, \beta_2]$	Adam optimizer parameters	[0.9, 0.999]

Supplementary File 1: **MNIST multicompartment network hyperparameters**. The hyperparameters used to train our multicompartment neural network model on the MNIST dataset.
