# Supplementary material for: Modeling the hallucinatory effects of classical psychedelics in terms of replay-dependent plasticity mechanisms
Source: eLife. 2026 Apr 21;14:RP105968. doi: 10.7554/eLife.105968 (PMC13099140; doi:10.7554/eLife.105968)
Supplement: Supplementary file 3. [file elife-105968-supp3.pdf]

Parameter	Function	Value
$\{N^{(l)}\}$	Layer Widths	$[512, 256, 256, 128, 64, 32, 16]$
σ_b	Denoised inference standard dev.	0.01
$\bar{\sigma}_b$	Noisy inference standard dev.	0.01
σ_p	Denoised generative standard dev.	0.01
$\bar{\sigma}_p$	Noisy generative standard dev.	0.01
L	Epoch number	400
T_w	Length of Wake phase	200
T_s	Length of Sleep phase	50
K	Batch number	512
η_p	Generative parameter learning rate	0.001
η_b	Inference parameter learning rate	0.0001
$[\beta_1, \beta_2]$	Adam optimizer parameters	[0.9, 0.999]

Supplementary File 3: **Recurrent network hyperparameters**. The hyperparameters used to train our recurrent neural network model on the MNIST dataset.
