# Supplementary material for: Modeling the hallucinatory effects of classical psychedelics in terms of replay-dependent plasticity mechanisms
Source: eLife. 2026 Apr 21;14:RP105968. doi: 10.7554/eLife.105968 (PMC13099140; doi:10.7554/eLife.105968)
Supplement: Supplementary file 4. [file elife-105968-supp4.pdf]

Supplementary File 4: Wake-Sleep Pseudocode

$e \leftarrow 0$
$\mathcal{L}_{prev} \leftarrow \infty$
while $e < L$ **do** ▷ Repeat for L data epochs
    for t in range$(0, T_w)$ **do** ▷ Wake Phase
        for k in range$(0, K)$ **do** ▷ Sample network states
            $\mathbf{r}^{(k)} \sim b(\mathbf{r}, \theta_b)$
            if epoch is complete **then** $e \leftarrow e + 1$
            end if
        end for
        $\mathcal{L}(\theta_p) \leftarrow \frac{1}{K}\sum_{k=0}^{K} -\log p(\mathbf{r}^{(k)}, \theta_p)$ ▷ Update generative parameters θ_p
        $\Delta\theta_p \leftarrow \eta_p \nabla_{\theta_p} \mathcal{L}(\theta_p)$
        $\theta_p \leftarrow \text{Adam}(\theta_p, \Delta\theta_p)$
    end for
    if $\mathcal{L}(\theta_p) < \mathcal{L}_{prev}$ **then**
        $\mathcal{L}_{prev} \leftarrow \mathcal{L}(\theta_p)$
        for t in range$(0, T_s)$ **do** ▷ Sleep Phase
            for k in range$(0, K)$ **do** ▷ Sample network states
                $\mathbf{r}^{(k)} \sim p(\mathbf{r}, \theta_p)$
            end for
            $\mathcal{L}(\theta_b) \leftarrow \frac{1}{K}\sum_{k=0}^{K} -\log b(\mathbf{r}^{(k)}, \theta_b)$ ▷ Update inference parameters θ_b
            $\Delta\theta_b \leftarrow \eta_b \nabla_{\theta_b} \mathcal{L}(\theta_b)$
            $\theta_b \leftarrow \text{Adam}(\theta_b, \Delta\theta_b)$
        end for
    end if
end while
