# Supplementary material for: Modeling the hallucinatory effects of classical psychedelics in terms of replay-dependent plasticity mechanisms
Source: eLife. 2026 Apr 21;14:RP105968. doi: 10.7554/eLife.105968 (PMC13099140; doi:10.7554/eLife.105968)
Supplement: Peer review history

# Editor report: eLife Assessment

**DOI:** 10.7554/eLife.105968.3.sa0

**Authors:** Anna C Schapiro

**Affiliations:** University of Pennsylvania United States

**Keywords:** Convincing, Useful

This paper provides a **useful** new theory of the hallucinatory effects of 5-HT2A psychedelics. The authors present **convincing** evidence that a computational model trained with the Wake-Sleep algorithm can reproduce some features of hallucinations by varying the strength of top-down connections in the model, though it is not clear that this model applies to 5-HT2A hallucinogens in particular. The work will be of interest to researchers studying hallucinations or offline activity and plasticity more broadly.

# Referee report: Reviewer #1 (Public review)

**DOI:** 10.7554/eLife.105968.3.sa1

Bredenberg et al. aim to model some of the visual and neural effects of psychedelics via the Wake-Sleep algorithm. This is an interesting study with findings that challenge certain mainstream ideas in psychedelic neuroscience.

While some of my concerns have been addressed in revision, I am still not convinced that this model applies to 5-HT2A hallucinogens, as opposed to a pharmacologically distinct hallucinogen. I think it is important to justify which class of hallucinogens this model applies to and distinguish it from other hallucinogens. While some researchers tend to group several hallucinogens together (e.g., 5-HT2A agonists, NMDA antagonists, kappa-opioids agonists), I'm not convinced this is warranted, when they have distinct subjective and cognitive effects (including quite different visual distortions, and again I point out that the kappa-opioid agonist salvinorin A, which is referred to as an "oneirogen," has been described as particularly dream-like, perhaps more so than 5-HT2A hallucinogens), as well as some differences in therapeutic outcomes (ketamine seems to not have as persisting of therapeutic effects, and kappa-opioid agonist have yet to be shown to be therapeutic). Their use patterns highlight this (e.g., 5-HT2A drugs are used less in non-festival/rave social settings compared to NMDA drugs like ketamine, which can be used frequently enough to the point of abuse; kappa-opioid agonists have quite mixed effects in terms of pleasurable outcomes, thereby rarely being used/abused and almost never to my knowledge being used recreationally).

In sum, more is needed to justify the claim that this work applies to 5-HT2A drugs in particular.

# Referee report: Reviewer #2 (Public review)

**DOI:** 10.7554/eLife.105968.3.sa2

This work is a nice contribution to the literature in articulating a specific, testable theory of how psychedelics act to generate hallucinations and plasticity.

I believe my concerns from the first round of review have been addressed in this version.

# Author comment: Author response

**DOI:** 10.7554/eLife.105968.3.sa3

**Authors:** Colin Bredenberg, Fabrice Normandin, Blake Richards, Guillaume Lajoie

**Affiliations:** Université de Montréal Montreal Canada; Mila - Quebec Artificial Intelligence Institute Montreal Canada; McGill University Montreal Canada; Mila - Quebec Artificial Intelligence Institute Montreal Canada

The following is the authors’ response to the original reviews.

First, we thank the reviewers for the valuable and constructive reviews. Thanks to these, we believe the article has been considerably improved. We have organized our response to address points that are relevant to both reviewers first, after which we address the unique concerns of each individual reviewer separately. We briefly paraphrase each concern and provide comments for clarification, outlining the precise changes that we have made to the text.

**Common Concerns (R1 & R2):**

Can you clarify how NREM and REM sleep relate to the oneirogen hypothesis?

Within the submission draft we tried to stay agnostic as to whether mechanistically similar replay events occur during NREM or REM sleep; however, upon a more thorough literature review, we think that there is moderately greater evidence in favor of Wake-Sleep-type replay occurring during REM sleep which is related to classical psychedelic drug mechanisms of action.

First, we should clarify that replay has been observed during both REM and NREM sleep, and dreams have been documented during both sleep stages, though the characteristics of dreams differ across stages, with NREM dreams being more closely tied to recent episodic experience and REM dreams being more bizarre/hallucinatory (see Stickgold et al., 2001 for a review). Replay during sleep has been studied most thoroughly during NREM sharp-wave ripple events, in which significant cortical-hippocampal coupling has been observed (Ji & Wilson, 2007). However, it is critical to note that the quantification methods used to identify replay events in the hippocampal literature usually focus on identifying what we term ‘episodic replay,’ which involves a near-identical recapitulation of neural trajectories that were recently experienced during waking experimental recordings (Tingley & Peyrach, 2020). In contrast, our model focuses on ‘generative replay,’ where one expects only a statistically similar reproduction of neural activity, without any particular bias towards recent or experimentally controlled experience. This latter form of replay may look closer to the ‘reactivation’ observed in cortex by many studies (e.g. Nguyen et al., 2024), where correlation structures of neural activity similar to those observed during stimulus-driven experience are recapitulated. Under experimental conditions in which an animal is experiencing highly stereotyped activity repeatedly, over extended periods of time, these two forms of replay may be difficult to dissociate.

Interestingly, though NREM replay has been shown to couple hippocampal and cortical activity, a similar study in waking animals administered psychedelics found hippocampal replay without any obvious coupling to cortical activity (Domenico et al., 2021). This could be because the coupling was not strong enough to produce full trajectories in the cortex (psychedelic administration did not increase ‘alpha’ enough), and that a causal manipulation of apical/basal influence in the cortex may be necessary to observe the increased coupling. Alternatively, as Reviewer 1 noted, it may be that psychedelics induce a form of hippocampus-decoupled replay, as one would expect from the REM stage of a recently proposed complementary learning systems model (Singh et al., 2022).

Evidence in favor of a similarity between the mechanism of action of classical psychedelics and the mechanism of action of memory consolidation/learning during REM sleep is actually quite strong. In particular, studies have shown that REM sleep increases the activity of soma-targeting parvalbumin (PV) interneurons and decreases the activity of apical dendrite-targeting somatostatin (SOM) interneurons (Niethard et al., 2021), that this shift in balance is controlled by higher-order thalamic nuclei, and that this shift in balance is critical for synaptic consolidation of both monocular deprivation effects in early visual cortex (Zhou et al., 2020) and for the consolidation of auditory fear conditioning in the dorsal prefrontal cortex (Aime et al., 2022). These last studies were not discussed in our previous text–we have added them, in addition to a more nuanced description of the evidence connecting our model to NREM and REM replay.

Relevant modifications: Page 4, 1st paragraph; Page 11, 1st paragraph.

Can you explain how synaptic plasticity induced by psychedelics within your model relates to learning at a behavioral level?

While the Wake-Sleep algorithm is a useful model for unsupervised statistical learning, it is not a model of reward or fear-based conditioning, which likely occur via different mechanisms in the brain (e.g. dopamine-dependent reinforcement learning or serotonin-dependent emotional learning). The Wake-Sleep algorithm is a ‘normative plasticity algorithm,’ that connects synaptic plasticity to the formation of structured neural representations, but it is not the case that all synaptic plasticity induced by psychedelic administration within our model should induce beneficial learning effects. According to the Wake-Sleep algorithm, plasticity at apical synapses is enhanced during the Wake phase, and plasticity at basal synapses is enhanced during the Sleep phase; under the oneirogen hypothesis, hallucinatory conditions (increased ‘alpha’) cause an increase in plasticity at both apical and basal sites. Because neural activity is in a fundamentally aberrant state when ‘alpha’ is increased, there are no theoretical guarantees that plasticity will improve performance on any objective: psychedelic-induced plasticity within our model could perhaps better be thought of as ‘noise’ that may have a positive or negative effect depending on the context.

In particular, such ‘noise’ may be beneficial for individuals or networks whose synapses have become locked in a suboptimal local minimum. The addition of large amounts of random plasticity could allow a system to extricate itself from such local minima over subsequent learning (or with careful selection of stimuli during psychedelic experience), similar to simulated annealing optimization approaches. If our model were fully validated, this view of psychedelic-induced plasticity as ‘noise’ could have relevance for efforts to alleviate the adverse effects of PTSD, early life trauma, or sensory deprivation; it may also provide a cautionary note against repeated use of psychedelic drugs within a short time frame, as the plasticity changes induced by psychedelic administration under our model are not guaranteed to be good or useful in-and-of themselves without subsequent re-learning and compensation.

We should also note that we have deliberately avoided connecting the oneirogen hypothesis model to fear extinction experimental results that have been observed through recordings of the hippocampus or the amygdala (Bombardi & Giovanni, 2013; Jiang et al., 2009; Kelly et al., 2024; Tiwari et al., 2024). Both regions receive extensive innervation directly from serotonergic synapses originating in the dorsal raphe nucleus, which have been shown to play an important role in emotional learning (Lesch & Waider, 2012); because classical psychedelics may play a more direct role in modulating this serotonergic innervation, it is possible that fear conditioning results (in addition to the anxiolytic effects of psychedelics) cannot be attributed to a shift in balance between apical and basal synapses induced by psychedelic administration. We have provided a more detailed review of these results in the text, as well as more clarity regarding their relation to our model.

Relevant modifications: Page 9, final paragraph; Page 12, final paragraph.

**Reviewer 1 Concerns:**

Is it reasonable to assign a scalar parameter ‘alpha’ to the effects of classical psychedelics? And is your proposed mechanism of action unique to classical psychedelics? E.g. Could this idea also apply to kappa opioid agonists, ketamine, or the neural mechanisms of hallucination disorders?

We have clarified that within our model ‘alpha’ is a parameter that reflects the balance between apical and basal synapses in determining the activity of neurons in the network. For the sake of simplicity we used a single ‘alpha’ parameter, but realistically, each neuron would have its own ‘alpha’ parameter, and different layers or individual neurons could be affected differentially by the administration of any particular drug; therefore, our scalar ‘alpha’ value can be thought of as a mean parameter for all neurons, disregarding heterogeneity across individual neurons.

There are many different mechanisms that could theoretically affect this ‘alpha’ parameter, including: 5-HT2a receptor agonism, kappa opioid receptor binding, ketamine administration, or possibly the effects of genetic mutations underlying the pathophysiology of complex developmental hallucination disorders. We focused exclusively on 5-HT2a receptor agonism for this study because the mechanism is comparatively simple and extensively characterized, but similar mechanisms may well be responsible for the hallucinatory symptoms of a variety of drugs and disorders.

Relevant modifications: Page 4, first paragraph; Page 13, first paragraph.

Can you clarify the role of 5-HT2a receptor expression on interneurons within your model?

While we mostly focused on the effects of 5-HT2a receptors on the apical dendrites of pyramidal neurons, these receptors are also expressed on soma-targeting parvalbumin (PV) interneurons. This expression on PV interneurons is consistent with our proposed psychedelic mechanism of action, because it could lead to a coordinated decrease in the influence of somatic and proximal dendritic inputs while increasing the influence of apical dendritic inputs. We have elaborated on this point, and moved the discussion earlier in the text.

Relevant modifications: Page 1, 1st paragraph; Page 4, 2nd paragraph.

Discussions of indigenous use of psychedelics over millenia may amount to over-romanticization.

We ultimately decided to remove these discussions from the main text, as they had little bearing on the content of our work. Within the Ethics Declarations section we softened our claims from “millenia” to “centuries,” as indigenous psychedelic use over this latter period of time is well-substantiated.

Relevant modifications: removed from introduction; modified Ethics Declarations

You isolate the 5-HT2a agonism as the mechanism of action underlying ‘alpha’ in your model, but there exist 5-HT2a agonists that do not have hallucinatory effects (e.g. lisuride). How do you explain this?

Lisuride has much-reduced hallucinatory effects compared to other psychedelic drugs at clinical doses (though it does indeed induce hallucinations at high doses; Marona-Lewicka et al., 2002), and we should note that serotonin (5-HT) itself is pervasive in the cortex without inducing hallucinatory effects during natural function. Similarly, MDMA is a partial agonist for 5-HT2a receptors, but it has much-reduced perceptual hallucination effects relative to classical psychedelics (Green et al., 2003) in addition to many other effects not induced by classical psychedelics.

Therefore, while we argue that 5-HT2a agonism induces an increase in influence of apical dendritic compartments and a decrease in influence of basal/somatic compartments, and that this change induces hallucinations, we also note that there are many other factors that control whether or not hallucinations are ultimately produced, so that not all 5-HT2a agonists are hallucinogenic. There are two possible additional factors that could contribute to this phenomenon: 5-HT receptor binding affinity and cellular membrane permeability.

Importantly, many 5-HT2a receptor agonists are also 5-HT1a receptor agonists (e.g. serotonin itself and lisuride), while MDMA has also been shown to increase serotonin, norepinephrine, and dopamine release (Green et al., 2003). While 5-HT2a receptor agonism has been shown to reduce sensory stimulus responses (Michaiel et al., 2019), 5-HT1a receptor agonism inhibits spontaneous cortical activity (Azimi et al., 2020); thus one might expect the net effect of administering serotonin or a nonselective 5-HT receptor agonist to be widespread inhibition of a circuit, as has been observed in visual cortex (Azimi et al., 2020). Therefore, selective 5-HT2a agonism is critical for the induction of hallucinations according to our model, though any intervention that jointly excites pyramidal neurons’ apical dendrites and inhibits their basal/somatic compartments across a broad enough area of cortex would be predicted to have a similar effect. Lisuride has a much higher binding affinity for 5-HT1a receptors than, for instance, LSD (Marona-Lewicka et al., 2002).

Secondly, it has recently been shown that both the head-twitch effect (a coarse behavioral readout of hallucinations in animals) and the plasticity effects of psychedelics are abolished when administering 5-HT2a agonists that are impermeable to the cellular membrane because of high polarity, and that these effects can be rescued by temporarily rendering the cellular membrane permeable (Vargas et al., 2023). This suggests that the critical hallucinatory effects of psychedelics (apical excitation according to our model) may be mediated by intracellular 5-HT2a receptors. Notably, serotonin itself is not membrane permeable in the cortex.

Therefore, either of these two properties could play a role in whether a given 5-HT2a agonist induces hallucinatory effects. We have provided an extended discussion of these nuances in our revision.

Relevant modifications: Page 1, paragraph 2.

Your model proposes that an increase in top-down influence on neural activity underlies the hallucinatory effects of psychedelics. How do you explain experimental results that show increases in bottom-up functional connectivity (either from early sensory areas or the thalamus)?

Firstly, we should note that our proposed increase in top-down influence is a causal, biophysical property, not necessarily a statistical/correlative one. As such, we will stress that the best way to test our model is via direct intervention in cortical microcircuitry, as opposed to correlative approaches taken by most fMRI studies, which have shown mixed results with regard to this particular question. Correlative approaches can be misleading due to dense recurrent coupling in the system, and due to the coarse temporal and spatial resolution provided by noninvasive recording technologies (changes in statistical/functional connectivity do not necessarily correspond to changes in causal/mechanistic connectivity, i.e. correlation does not imply causation).

There are two experimental results that appear to contradict our hypothesis that deserve special consideration. The first shows an increase in directional thalamic influence on the distributed cortical networks after psychedelic administration (Preller et al., 2018). To explain this, we note that this study does not distinguish between lower-order sensory thalamic nuclei (e.g. the lateral and medial geniculate nuclei receiving visual and auditory stimuli respectively) and the higher-order thalamic nuclei that participate in thalamocortical connectivity loops (Whyte et al., 2024). Subsequent more fine-grained studies have noted an increase in influence of higher order thalamic nuclei on the cortex (Pizzi et al., 2023; Gaddis et al., 2022), and in fact extensive causal intervention research has shown that classical psychedelics (and 5-HT2a agonism) decrease the influence of incoming sensory stimuli on the activity of early sensory cortical areas, indicating decoupling from the sensory thalamus (Evarts et al., 1955; Azimi et al., 2020; Michaiel et al. 2019). The increased influence of higher-order thalamic nuclei is consistent with both the cortico-striatal-thalamo-cortical (CTSC) model of psychedelic action as well as the oneirogen hypothesis, since higher-order thalamic inputs modulate the apical dendrites of pyramidal neurons in cortex (Whyte et al., 2024).

The second experimental result notes that DMT induces traveling waves during resting state activity that propagate from early visual cortex to deeper cortical layers (Alamia et al., 2020). There are several possibilities that could explain this phenomenon: (1) it could be due to the aforementioned difficulties associated with directed functional connectivity analyses, (2) it could be due to a possible high binding affinity for DMT in the visual cortex relative to other brain areas, or (3) it could be due to increases in apical influence on activity caused by local recurrent connectivity within the visual cortex which, in the absence of sensory input, could lead to propagation of neural activity from the visual cortex to the rest of the brain. This last possibility is closest to the model proposed by (Ermentrout & Cowan, 1979), and which we believe would be best explained within our framework by a topographically connected recurrent network architecture trained on video data; a potentially fruitful direction for future research.

Relevant modifications: Page 9, paragraph 1; Page 10, final paragraph; Page 11, final paragraph.

Shouldn’t the hallucinations generated by your model look more ‘psychedelic,’ like those produced by the DeepDream algorithm?

We believe that the differences in hallucination visualization quality between our Wake-Sleep-trained models and DeepDream are mostly due to differences in the scale and power of the models used across these two studies. We are confident that with more resources (and potentially theoretical innovations to improve the Wake-Sleep algorithm’s performance) the produced hallucination visualizations could become more realistic.

We note that more powerful generative models trained with backpropagation are able to produce surreal images of comparable quality (Rezende et al., 2014; Goodfellow et al., 2020; Vahdat & Kautz, 2020), though these have not yet been used as a model of psychedelic hallucinations. However, the DeepDream model operates on top of large pretrained image processing models, and does not provide an biologically mechanistic/testable interpretation of its hallucination effects. When training smaller models with a local synaptic plasticity rule (as opposed to backpropagation), the hallucination effects are less visually striking due to the reduced quality of our trained generative model, though they are still strongly tied to the statistics of sensory inputs, as quantified by our correlation similarity metric (Fig. 5b).

To demonstrate that our proposed hallucination mechanism is capable of producing more complex hallucinations in larger, more powerful models, we employed our same hallucination generation mechanism in a pretrained Very Deep Variational Autoencoder (VDVAE) (Child et al., 2021), which is a hierarchical variational autoencoder with a nearly identical structure compared to our Wake-Sleep-trained networks, with both a bottom-up inference pathway and a top-down generative pathway that maps cleanly onto our multicompartmental neuron model. VDVAEs are trained on the same objective function as our Wake-Sleep-trained networks, but using the backpropagation algorithm. The VDVAE models were able to generate much more complex hallucinations (emergence of complex geometric patterns, smooth deformations of objects and faces), whose complexity arguably exceeds those produced by the DeepDream algorithm. Therefore while the VDVAEs are less biologically realistic (they do not learn via local synaptic plasticity), they function as a valuable high-level model of hallucination generation that complements our Wake-Sleep-trained approach. As further validation, we were also able to replicate our key results and testable predictions with these models.

Relevant modifications: Results section “Modeling hallucinations in large-scale pretrained networks”; Figure 6, S7, S8; Page 12, paragraph 3; Methods section “Generating hallucinations in hierarchical variational autoencoders.”

Your model assumes domination by entirely bottom-up activity during the ‘wake’ phase, and domination entirely by top-down activity during ‘sleep,’ despite experimental evidence indicating that a mixture of top-down and bottom-up inputs influence neural activity during both stages in the brain. How do you explain this?

Our use of the Wake-Sleep algorithm, in which top-down inputs (Sleep) or bottom-up inputs (Wake) dominate network activity is an over-simplification made within our model for computational and theoretical reasons. Models that receive a mixture of top-down and bottom-up inputs during ‘Wake’ activity do exist (in particular the closely related Boltzmann machine (Ackley et al., 1985)), but these models are considerably more computationally costly to train due to a need to run extensive recurrent network relaxation dynamics for each input stimulus. Further, these models do not generalize as cleanly to processing temporal inputs. For this reason, we focused on the Wake-Sleep algorithm, at the cost of some biological realism, though we note that our model should certainly be extended to support mixed apical-basal waking regimes. We have added a discussion of this in our ‘Model Limitations’ section.

Relevant modifications: Page 12, paragraph 4.

Your model proposes that 5-HT2a agonism enhances glutamatergic transmission, but this is not true in the hippocampus, which shows decreases in glutamate after psychedelic administration.

We should note that our model suggests only compartment specific increases in glutamatergic transmission; as such, our model does not predict any particular directionality for measures of glutamatergic transmission that includes signaling at both apical and basal compartments in aggregate, as was measured in the provided study (Mason et al., 2020).

You claim that your model is consistent with the Entropic Brain theory, but you report increases in variance, not entropy. In fact, it has been shown that variance decreases while entropy increases under psychedelic administration. How do you explain this discrepancy?

Unfortunately, ‘entropy’ and ‘variance’ are heavily overloaded terms in the noninvasive imaging literature, and the particularities of the method employed can exert a strong influence on the reported effects. The reduction in variance reported by (Carhart-Harris et al., 2016) is a very particular measure: they are reporting the variance of resting state synchronous activity, averaged across a functional subnetwork that spans many voxels; as such, the reduction in variance in this case is a reduction in broad, synchronous activity. We do not have any resting state synchronous activity in our network due to the simplified nature of our model (particularly an absence of recurrent temporal dynamics), so we see no reduction in variance in our model due to these effects.

Other studies estimate ‘entropy’ or network state disorder via three different methods that we have been able to identify. (1) (Carhart-Harris et al., 2014) uses a different measure of variance: in this case, they subtract out synchronous activity within functional subnetworks, and calculate variability across units in the network. This measure reports increases in variance (Fig. 6), and is the closest measure to the one we employ in this study. (2) (Lebedev et al., 2016) uses sample entropy, which is a measure of temporal sequence predictability. It is specifically designed to disregard highly predictable signals, and so one might imagine that it is a measure that is robust to shared synchronous activity (e.g. resting state oscillations). (3) (Mediano et al., 2024) uses Lempel-Ziv complexity, which is, similar to sample entropy, a measure of sequence diversity; in this case the signal is binarized before calculation, which makes this method considerably different from ours. All three of the preceding methods report increases in sequence diversity, in agreement with our quantification method. Our strongest explanation for why the variance calculation in (Carhart-Harris et al., 2016) produces a variance reduction is therefore due to a reduction in low-rank synchronous activity in subnetworks during resting state.

As for whether the entropy increase is meaningful: we share Reviewer 1’s concern that increases in entropy could simply be due to a higher degree of cognitive engagement during resting state recordings, due to the presence of sensory hallucinations or due to an inability to fall asleep. This could explain why entropy increases are much more minimal relative to non-hallucinating conditions during audiovisual task performance (Siegel et al., 2024; Mediano et al., 2024). However, we can say that our model is consistent with the Entropic Brain Theory without including any form of ‘cognitive processing’: we observe increases in variability during resting state in our model, but we observe highly similar distributions of activity when averaging over a wide variety of sensory stimulus presentations (Fig. 5b-c). This is because variability in our model is not due to unstructured noise: it corresponds to an exploration of network states that would ordinarily be visited by some stimulus. Therefore, when averaging across a wide variety of stimuli, the distribution of network states under hallucinating or non-hallucinating conditions should be highly similar.

One final point of clarification: here we are distinguishing Entropic Brain Theory from the REBUS model–the oneirogen hypothesis is consistent with the increase in entropy observed experimentally, but in our model this entropy increase is not due to increased influence of bottom-up inputs (it is due instead to an increase in top-down influence). Therefore, one could view the oneirogen hypothesis as consistent with EBT, but inconsistent with REBUS.

Relevant modifications: Page 10, paragraph 1.

You relate your plasticity rule to behavioral-timescale plasticity (BTSP) in the hippocampus, but plasticity has been shown to be reduced in the hippocampus after psychedelic administration. Could you elaborate on this connection?

When we were establishing a connection between our ‘Wake-Sleep’ plasticity rule and BTSP learning, the intended connection was exclusively to the mathematical form of the plasticity rule, in which activity in the apical dendrites of pyramidal neurons functions as an instructive signal for plasticity in basal synapses (and vice versa): we will clarify this in the text. Similarly, we point out that such a plasticity rule tends to result in correlated tuning between apical and basal dendritic compartments, which has been observed in hippocampus and cortex: this is intended as a sanity check of our mapping of the Wake-Sleep algorithm to cortical microcircuitry, and has limited further bearing on the effects of psychedelics specifically.

Reduction in plasticity in the hippocampus after psychedelic administration could be due to a complementary learning systems-type model, in which the hippocampus becomes partly decoupled from the cortex during REM sleep (Singh et al., 2022); were this to be the case, it would not be incompatible with our model, which is mostly focused on the cortex. Notably, potentiating 5HT-2a receptors in the ventral hippocampus does not induce the head-twitch response, though it does produce anxiolytic effects (Tiwari et al., 2024), indicating that the hallucinatory and anxiolytic effects of classical psychedelics may be partly decoupled.

**Reviewer 2 Concerns:**

Could you provide visualizations of the ‘ripple’ phenomenon that you’re referring to?

In our revised submission, ‘ripple’ phenomena are now visible in two places: Fig 2c-d, and Fig 6 (rows 2 and 3). Because the VDVAE models used to generate Figure 6 produce higher quality generated images, the ripples appearing in these plots are likely more prototypical, but it is not easy to evaluate the quality of these visualizations relative to subjective hallucination phenomena.

Could you provide a more nuanced description of alternative roles for top-down feedback, beyond being used exclusively for learning as depicted in your model?

For the sake of simplicity, we only treat top-down inputs in our model as a source of an instructive teaching signal, the originator of generative replay events during the Sleep phase, and as the mechanism of hallucination generation. However, as discussed in a response to a previous question, in the cortex pyramidal neurons receive and respond to a mixture of top-down and bottom-up processing.

There are a variety of theories for what role top-down inputs could play in determining network activity. To name several, top-down input could function as: (1) a denoising/pattern completion signal (Kadkhodaie & Simoncelli, 2021), (2) a feedback control signal (Podlaski & Machens, 2020), (3) an attention signal (Lindsay, 2020), (4) ordinary inputs for dynamic recurrent processing that play no specialized role distinct from bottom-up or lateral inputs except to provide inputs from higher-order association areas or other sensory modalities (Kar et al., 2019; Tugsbayar et al., 2025). Though our model does not include these features, they are perfectly consistent with our approach.

In particular, denoising/pattern completion signals in the predictive coding framework (closely related to the Wake-Sleep algorithm) also play a role as an instructive learning signal (Salvatori et al., 2021); and top-down control signals can play a similar role in some models (Gilra & Gerstner, 2017; Meulemans et al., 2021). Thus, options 1 and 2 are heavily overlapping with our approach, and are a natural consequence of many biologically plausible learning algorithms that minimize a variational free energy loss (Rao & Ballard, 1997; Ackley et al., 1985). Similarly, top-down attentional signals can exist alongside top-down learning signals, and some models have argued that such signals can be heavily overlapping or mutually interchangeable (Roelfsema & van Ooyen, 2005). Lastly, generic recurrent connectivity (from any source) can be incorporated into the Wake-Sleep algorithm (Dayan & Hinton, 1996), though we avoided doing this in the present study due to an absence of empirical architecture exploration in the literature and the computational complexity associated with training on time series data.

To conclude, there are a variety of alternative functions proposed for top-down inputs onto pyramidal neurons in the cortex, and we view these additional features as mutually compatible with our approach; for simplicity we did not include them in our Wake-Sleep-trained model, but we believe that these features are unlikely to interfere with our testable predictions or empirical results. In fact, the pretrained VDVAE models that we worked with do include top-down influence during the Wake-stage inference process, and these models recapitulated our key results and testable predictions (Fig. S8).

Relevant modifications: Fig. S8; Page 12, paragraph 4.
